# Intrathecal Injection of Hyperbaric Bupivacaine Versus a Mixture of Hyperbaric and Isobaric Bupivacaine in Lower Abdominal Surgery: A Randomized Controlled Trial

**DOI:** 10.5812/aapm-142719

**Published:** 2024-01-03

**Authors:** Adel Ali Hassan, Amira Seleem Saleh, Maged Salah Mohamed, Moataz Salah Khalil

**Affiliations:** 1 Anesthesia, Surgical Intensive Care and Pain Management Department, Faculty of Medicine, Helwan University, Helwan, Egypt; 2 Anesthesia, Surgical Intensive Care and Pain Management Department, Faculty of Medicine, Cairo University, Cairo, Egypt

**Keywords:** Intrathecal, Hyperbaric, Isobaric, Bupivacaine

## Abstract

**Background:**

Bupivacaine hydrochloride is widely used as the primary drug for spinal anesthesia.

**Objectives:**

This research aimed to evaluate the intrathecal administration of both isobaric and hyperbaric bupivacaine (HB) in lower abdominal surgery.

**Methods:**

A randomized, controlled, double-blind trial was conducted on 50 patients classified as American Society of Anesthesiologists (ASA) class I to II, scheduled for lower abdominal surgery under spinal anesthesia. The patients were allocated randomly into two groups of similar size. Group A (control group) received 20 mg HB 0.5% intrathecally. Group B (case group) received 10 mg HB 0.5% and 10 mg isobaric bupivacaine (IB) 0.5%.

**Results:**

There was a significant decline in heart rate and mean arterial pressure in Group A compared to Group B (P < 0.05). Group A had a significantly greater sensory level at 10 and 20 minutes than Group B (P = 0.008 and 0.006, respectively). Group A had an earlier duration in reaching Bromage 3 and the first need for analgesia, compared to group B (P = 0.001 and 0.003, respectively).

**Conclusions:**

In lower abdominal surgery, the intrathecal administration of HB with IB increased hemodynamic stability and duration of both sensory and motor blockade but with slower recovery from anesthesia compared to HB alone.

## 1. Background

Because of its rapid onset and cost-effectiveness, spinal anesthesia (SA) is one of the most often utilized techniques for surgical procedures in the lower abdomen, perineum, and lower extremities (1). The benefits of SA are its modest dosage requirements, ease of administration, quick onset time, consistent operative analgesia, and effective muscular relaxation (2).

A solution's baricity is determined by dividing its density by the density of the cerebrospinal fluid (CSF) (3). Bupivacaine is the most commonly used anesthetic for SA in non-obstetric and obstetric surgery (4). It may be prepared as an isobaric or hyperbaric solution. Alterations in the baricity of spinal anesthetic solutions influence the distribution and hemodynamic parameters in the subarachnoid space (5, 6). This may influence the onset, duration, and severity of sensory block and any side effects (4, 6).

Many side effects have been associated with SA, the most frequent one being hypotension. In the general population, 25% to 75% of cases develop hypotension. Various variables contribute to an increased susceptibility to hypotension, including both patient-related and technical aspects (7, 8). Patient-related factors include advanced age, pregnancy, obesity, diabetes mellitus (DM), anemia, and hypertension. On the other hand, technical considerations include the presence of a block level at or above T5, administration of large doses of local anesthetics, and use of opioids during premedication (9, 10).

All anesthesia professionals aim to perform an appropriate level of SA with a lower incidence of complications; in doing so, regional anesthetics of various baricity are used (11).

There is a prevailing belief that hyperbaric solutions are more optimal for accessing the upper thoracic dermatomes than their plain equivalents. The primary objective of anesthesia experts is to provide SA at a suitable level while minimizing complications. Regional anesthetics with various baricity are used (6).

## 2. Objectives

This research aimed to compare intrathecal administration of hyperbaric bupivacaine (HB) vs. isobaric and HB in lower abdominal surgery.

## 3. Methods

This randomized, controlled, double-blind trial was conducted on 50 patients from May 2022 to July 2023.

### 3.1. Inclusion Criteria

Eighteen to 56-year-old patients from both sexes classified according to the American Society of Anesthesiologists (ASA) as class I to II who were scheduled for lower abdominal surgery under spinal anesthesia were included. The research was conducted after the consent of the Ethical Committee of Helwan University Hospitals, Helwan, Egypt (Code: 48-2022). All patients provided informed written consent. The trial was registered on clinicaltrials.gov (ID: NCT06050044).

### 3.2. Exclusion Criteria

Cases with amide allergies, drug abuse, diabetes mellitus (DM), neurological or neuromuscular diseases, cardiovascular illnesses, and pregnant individuals were excluded.

### 3.3. Randomization and Blindness

Concealing the assignment was accomplished using sealed opaque envelopes. Both outcome assessors and patients were blinded by relevant information throughout the research. The administration of SA was performed by an anesthesiologist who did not participate in the research. The patients were allocated randomly into two equal groups of similar size. Group A (control group) received 20 mg HB 0.5% intrathecally, and Group B (case group) received 10 mg HB 0.5% and 10 mg isobaric bupivacaine (IB) 0.5%.

Monitoring by pulse oximetry, noninvasive blood pressure, and ECG was implemented. An intravenous (IV) cannula was inserted. A preload of 500 mL of 0.9% sodium chloride solution was administered. Conscious sedation was achieved using IV midazolam at a dosage range of 0.01 to 0.05 mg/kg in all subjects before SA administration.

SA was given to all subjects while sitting with a 27-gauge Quincke spinal needle through the L4 - L5 spaces using a midline approach and standard aseptic conditions. The tip of the needle was directed in the cephalad direction. Group A was injected with 4 mL of HB and while group B was injected with 2 mL of hyperbaric bupivacaine followed by 2 mL of the isobaric form. The injection of local anesthetic solutions was done gradually over a period of 30 seconds.

The same researcher used the modified Bromage scale to check on the motor block every five minutes (1) (1: Complete movement, 2: Unable to flex the hips, can bend the knee, 3: Unable to flex knee yet able to flex the ankle and 4: No movement). The times needed to reach Bromage 3 before surgery and regress to Bromage 0 after surgery were recorded.

The assessment of the sensory block at the segmental level was conducted bilaterally using a cold applicator. The sensory block levels were assessed at 2 and 5 minutes after injection and at five-minute intervals until two successive sensory block levels were similar. Patients who failed to achieve a sensory block reaching the T6 level within twenty minutes were excluded from participation in the trial. Patients describing discomfort received intravenous injections of fentanyl 50 μg, but SA converted to general anesthesia after two boluses. The time to first analgesia requirement was recorded. A ten-point Visual Analog Scale (VAS) was used to assess the intensity of their pain severity.

Heart rate (HR) and mean arterial pressure (MAP) were measured at baseline 5, 10, 15, 20, 25, 30, 45, 60 and 120 minutes after the injection.

Post-operative complications were recorded, such as hypotension (defined as MAP <65 mmHg or decrease than basal MAP by 20% and was treated with IV fluid), bradycardia (defined as HR < 50 beats/min and was treated by IV atropine 0.02 mg/kg), shivering (treated by pethidine 30 mg IV bolus), nausea, vomiting (treated by IV ondansetron 4mg) and discomfort.

The primary outcome was the occurrence of hypotension. The secondary outcomes were hemodynamic parameters, sensory and motor levels and duration, and the need for post-operative analgesic medication.

### 3.4. Sample Size Calculation

Cesur et al. (12) revealed a higher occurrence of hypotension, namely 66.7%, while using HB compared to a lower incidence of 13.9% observed using isobaric and HB. This resulted in a risk ratio of 4.7. By using the Openepi online software, it was calculated that to obtain a power of 90%, a minimum sample size of 25 cases per group was required. This analysis was conducted using Fisher’s exact test, with a threshold of significance set at 0.05. The sample size was increased by 20% to account for the dropouts.

### 3.5. Statistical Analysis 

The statistical analysis was conducted using SPSS v26 (IBM Inc. in Chicago, IL, USA). The standard deviation (SD) and mean were used to represent the quantitative variables, and an unpaired Student's *t*-test was used to compare these variables between the two groups. Repeated measure ANOVA was used to compare multiple endpoints with Bonferroni corrections for multiple comparisons. The qualitative variables were represented in percentage and frequency and were subjected to analysis using either Fisher's exact test or the chi-square test, as deemed suitable. A two-tailed P value less than 0.05 was considered statistically significant.

## 4. Results

**Figure 1.**
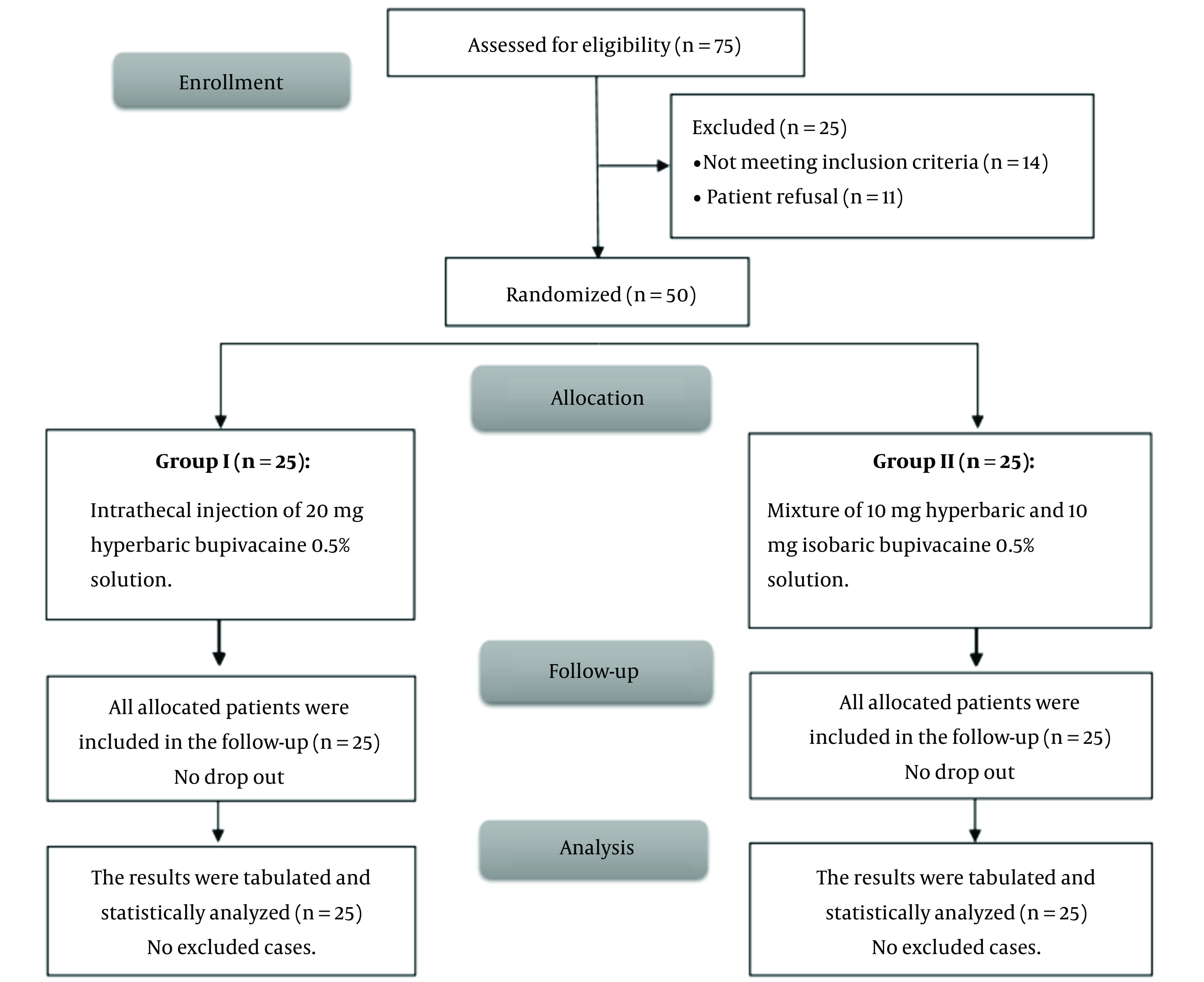
CONSORT flowchart of the enrolled patients.

This research evaluated 75 patients for eligibility; fourteen did not meet the criteria, and eleven refused to participate. The remaining patients were randomly allocated into two equal groups (25 patients each). All allocated patients were analyzed and followed up statistically (Figure 1). 

There were no significant differences between the groups regarding patient characteristics and duration of operation (Table 1). 

**Table 1.** Patient Characteristics and Duration of Operations of Studied Groups ^a^

Variables	Group A (N = 25)	Group B (N = 25)	P-Value
**Age (y)**	38.4 ± 12.28	38.32 ± 10.64	0.980
**Sex**			0.556
Male	17 (68)	15 (60)	
Female	8 (32)	10 (40)	
**Weight (kg)**	69.8 ± 10.43	69.32 ± 8.09	0.856
**Height (cm)**	166.76 ± 6.74	167.72 ± 6.01	0.598
**BMI (kg/m** ^**2**^ **)**	25.25 ± 4.42	24.74 ± 3.41	0.649
**ASA physical status**			0.99
I	22 (88.0)	21 (84.0)	
II	3 (12.0)	4 (16.0)	
**Types of operations**			0.394
Inguinal hernia	5 (20)	6 (24)	
Piles	10 (40)	4 (16)	
URS	6 (24)	8 (32)	
Pilonidal sinus	1 (4)	3 (12)	
TAH	3 (12)	4 (16)	
**Duration of operation (min)**	80.2 ± 18.73	83.8 ± 19.96	0514

Abbreviations: BMI, body mass index; ASA, American Society of Anesthesiologists; URS, ureterorenoscopy; TAH, total abdominal hysterectomy.

^a^ Values are presented as No. (%) or mean ± SD.

**Figure 2.**
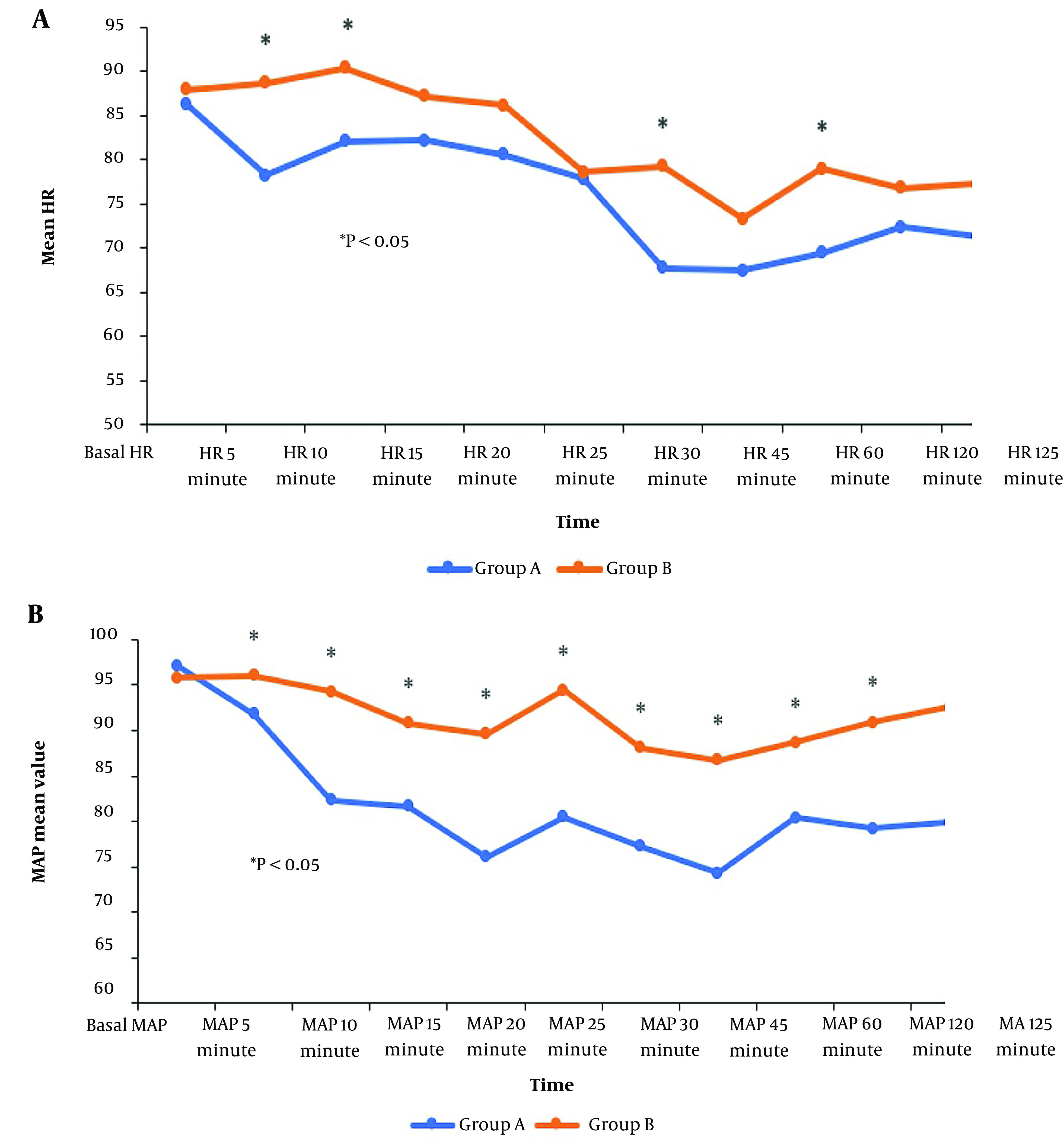
Mean arterial blood pressure (MAP) and heart rate (HR) values in both groups

There was a significant decrease in HR at 5, 10, 30, 60, and 180 minutes and MAP at 5 to 180 minutes in Group A compared to Group B (P < 0.05) Figure 2. 

There was a significantly shorter time to reach Bromage 3 and the first analgesia requirement in group A than in group B (P < 0.05). There was no difference regarding the time to first movement and time to reach two consecutive levels in both groups. Regarding sensory level at serial times, there was a significantly higher anesthetic effect in group A at ten to twenty minutes compared to group B (P < 0.05) (Table 2). 

**Table 2.** Time to “Reach Bromage 3, First Movement, Reach Two Consecutive Level and First Analgesia Requirement” and Sensory Level at Serial Times in Studied Groups ^a^

Variables	Group A (N = 25)	Group B (N = 25)	P-Value
**Time to reach Bromage 3 **	4.64 ± 1.47	6.12 ± 1.54	0.001^b^
**Time to first movement (min)**	131.84 ± 22.56	134.8 ± 22.84	0.647
**Time to reach two consecutive levels (min)**	14.8 ± 3.38	13.2 ± 2.45	0.062
**Time to first analgesia requirement (min)**	175 ± 19.84	196.6 ± 28.38	0.003^b^
**Sensory level at serial times**			
2 minutes	10.8 ± 1	11.04 ± 1.02	0.405
5 minutes	10.2 ± 0.66	9.92 ± 1.68	0.884
10 minutes	9.04 ± 1.74	7.52 ± 2.1	0.008^b^
15 minutes	6.48 ± 1.66	5.36 ± 0.95	0.006^b^
20 minutes	5.84 ± 0.55	5.28 ± 0.98	0.017^b^

^a^ Values are presented as mean ± SD.

^b^ significant P value.

**Figure 3.**
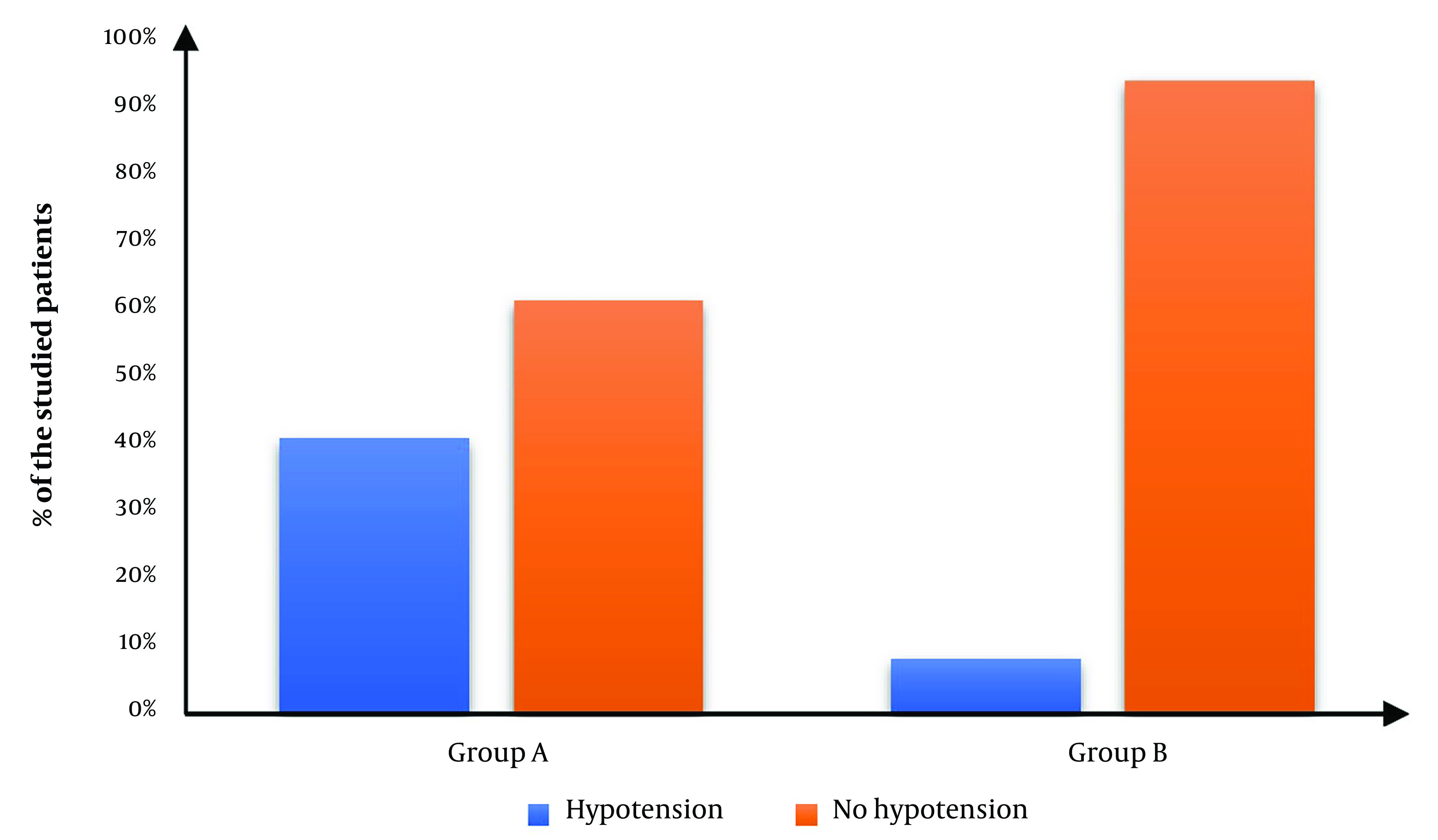
Hypotension of the studied groups.

Hypotension occurred in 10 (40%) patients in group A and in 2 (8%) in group B with RR (95% CI) 2.11 (1.322:3.371). There was a significantly lower occurrence of hypotension in group B than in group A (P value = 0.018) (Table 3, Figure 3). 

**Table 3.** Comparison of Complications of Two Modalities ^a^

	Group A (N = 25)	Group B (N = 25)	P-Value	RR (95% CI)
**Hypotension**	10 (40)	2 (8)	0.018^b^	2.11 (1.322:3.371)
**Bradycardia**	1 (4.0)	0 (0)	0.99	2.04 (1.534:2.717)
**Shivering**	5 (20.0)	2 (8.0)	0.42	1.53 (0.870:2.710)
**Nausea**	2 (8.0)	1 (4.0)	0.99	1.36 (0.5811:3.194)
**Discomfort**	1 (4.0)	1 (4.0)	1	1 (0.243:4.116)

Abbreviations: RR, relative risk; CI, confidence interval.

^a^ Values are presented as No. (%).

^b^ significant P-value.

The groups had no significant difference in shivering, nausea, bradycardia, and discomfort (Table 3). 

## 5. Discussion

It has been well acknowledged that modifying the anesthetic profiles may be achieved by including dextrose to enhance the specific gravity of local anesthetic solutions (13-15).

Bupivacaine hydrochloride is an aminoacyl local anesthetic and is the most commonly used local anesthetic medication for SA. There are two forms of commercially available bupivacaine: IB, with a density equal to that of CSF, and HB, with a density heavier than CSF. The difference in densities of the two available preparations is believed to affect their diffusion patterns and thus determine the drug's effectiveness, spread (dermatome height or block height), and side-effect profile (16).

Our present results regarding HR and MAP revealed that in group A, they decreased to 5 minutes, then elevated to 10 and 15 minutes, and then slowly decreased from 20 minutes to 3 hours. The stress of SA administration may explain the brief rise. In group B, they increased the duration to 5 and 10 minutes, then slowly decreased it from 15 minutes to 3 hours. There was a significant decrease in group A compared to group B.

These findings were compatible with Solakovic (17), who documented notable alterations in fundamental hemodynamic parameters after the administration of anesthesia, specifically with decreased blood pressure and slowdown of HR. The observed alterations in the hyperbaric group exhibited considerably greater magnitudes than the isobaric group across all assessed parameters. Upadya et al. (18) aimed to examine the hemodynamic impact of two different mixtures, namely intrathecal IB-fentanyl (group one) and HB -fentanyl (group two), during routine urological operations. It was observed that comparing the mean HR values among the two groups did not provide any statistically important findings. Nevertheless, it is important to highlight the use of adjuvant medications, the variations in techniques employed for regional anesthetic, and the variety in dosage in this study. Our findings demonstrated a more stable hemodynamic profile, with a significantly lower incidence of hypotension in the isobaric hyperbaric mixture group. This emphasizes the importance of the application of variant anesthetic baricity. Similar findings were noted by Alrefaey and Bakrey (19), who observed a significant decline in MAP 5 minutes after administering an intrathecal injection of three mL of 0.5% HB combined with 15 µg of fentanyl in old patients (age > 60) scheduled for lower limb orthopedic surgery. Also, Cesur et al. (12) showed that the sequential subarachnoid injection of hyperbaric and ordinary bupivacaine in cesarean section resulted in a significant reduction in the occurrence of hypotension (66.7% vs. 13.9%) as compared to HB. 

Gupta et al. (20) demonstrated that in some cases, a dosage of three mL of isobaric ropivacaine and 3 mL of hyperbaric ropivacaine in other cases, with a concentration of 6 mg/mL, resulted in a total dose of 18 mg. All cases exhibited hemodynamic stability during the surgical procedure, but this observation was not consistent with our current research findings.

Our research findings demonstrated a significantly shorter time to reach Bromage 3 in the hyperbaric group compared to the mixture group. This can be attributed to the restricted spread of HB in the CSF. There was no significant difference regarding time to first movement in both groups. Similar findings were obtained by Helmi et al. (21), who observed that the onset of motor block showed a shorter time in the hyperbaric group than the isobaric one. Regarding the duration of the motor block, it was longer in the isobaric group when compared to the hyperbaric group. A study by Kumar (22) showed that the initiation of motor blockade was quicker when using IB, which contradicts our findings. Furthermore, the duration of analgesia was extended with the use of IB, confirming our findings. The demographic differences in the patient population and different doses of drugs used may have contributed to this difference.

Our findings revealed no significant difference regarding the time to reach two consecutive levels in both groups. Still, the hyperbaric group had a significantly shorter time to first analgesia requirement than the hyperbaric and isobaric mixture group. Because of the restricted diffusion of IB compared to HB, we hypothesize that the longer duration of sensory block is associated with greater concentration retained at the injection site.

Our findings demonstrated that sensory levels in the hyperbaric group two minutes after injection ranged between T10 - T12 and increased gradually at 20 minutes. The sensory level in the mixture group 2 minutes after spinal administration ranged between T10 - T12 and rose gradually from 5 to 20 minutes. A significantly higher sensory level was observed in patients who received HB only, with less analgesia time than the other group.

This is consistent with a previous study by Gupta et al. (20), who reported that using hyperbaric ropivacaine caused a quicker start of sensory block, which in turn caused a quicker regress. A study by Kumar (22) demonstrated that in the hyperbaric and isobaric groups, the average periods to seek rescue analgesia were 308.6 ± 14.9 minutes and 365.9 ± 12.3 minutes, respectively. This confirms our results.

While other studies reported that isobaric solutions may reach higher levels with a lesser duration of action, Upadya et al. (18) showed that the motor and sensory block duration was significantly lower in the isobaric group compared to the hyperbaric group. 

Also, Helmi et al. (21) demonstrated that the initiation of sensory blockage was higher in the isobaric than in the hyperbaric solution. The highest level of dermatome block was in thoracal 4, while the lowest was in Th 10. Most block levels were in thoracal 6 or 7 in Group I, while Group H produced lower blockade at Th 8 to Th 10.

We found that the difference in the occurrence of complications was statistically insignificant. However, there was a significant decrease in hypotension in Group B compared to Group A. This was compatible with the findings of Helmi et al. (21), who reported that hypotension occurred in more patients in the isobaric group than in the hyperbaric group. At the same time, the other adverse events (bradycardia and nausea) were comparable for both groups. However, Upadya et al. (18) observed that the hyperbaric group had a higher occurrence of postoperative shivering, bradycardia, and hypotension than the isobaric group.

This study confirmed our hypothesis that combining both drugs would enhance their benefits while reducing undesirable side effects. 

Limitations of the study included a relatively small number of patients at one single location. Furthermore, using the research at a teaching hospital extends the surgery time beyond three hours. However, given the associated heavier block and hemodynamic effects, we believe that this would increase the significance of the published data. Moreover, the volume of CSF was not determined in the spine, while the height of a patient would affect the sensory level and the findings of this research.

### 5.1. Conclusions

In lower abdominal surgery, administering hyperbaric with IB by intrathecal injection increased hemodynamic stability and sensory and motor blockade duration but with slower recovery from anesthesia compared to the administration of HB alone.

## Data Availability

The dataset presented in the study is available on request from the corresponding author during submission or after publication.

## Clinical Trial Registration Code:

NCT06050044.
